# Diabetes Treatment Is Associated With Better Cognitive Function: The Age Disparity

**DOI:** 10.3389/fnagi.2021.753129

**Published:** 2022-01-06

**Authors:** Keyi Wu, Huamin Liu, Jiazhen Zheng, Lianwu Zou, Shanyuan Gu, Rui Zhou, Zelin Yuan, Zhiwei Huang, Xianbo Wu

**Affiliations:** ^1^Department of Epidemiology, School of Public Health, Southern Medical University (Guangdong Provincial Key Laboratory of Tropical Disease Research), Guangzhou, China; ^2^Department of Psychiatry, Baiyun Jingkang Hospital, Guangzhou, China; ^3^Inpatient Department, Baiyun Jingkang Hospital, Guangzhou, China

**Keywords:** diabetes, cognitive decline, epidemiology, antidiabetic treatment, ageing

## Abstract

**Background:** Diabetes mellitus (DM) is a recognised risk factor for cognitive dysfunction. The purpose of this study was to explore the relationship between active treatment for DM and cognitive function in middle-aged (< 60 years) and older adults (≥60 years), respectively.

**Methods:** A total of 13,691 participants (58.55 ± 9.64 years, 47.40% of men) from the Chinese Health and Retirement Longitudinal Study (CHARLS) were included. The participants were classified into three groups according to whether or not they have diabetes and to their diabetes treatment status: diabetes-free, treated-diabetes and untreated-diabetes, in which the diabetes-free group was regarded as reference specially. Cognitive function was assessed by two interview-based measurements for mental intactness and episodic memory.

**Results:** Compared with the participants in the diabetes-free group, the older participants in the treated-diabetes group had better performance in terms of mental intactness (β = 0.37, 95% CI = 0.04–0.70). No significant association was observed in the middle-aged participants. In the subgroup analyses, the lower cognitive score was only observed in people without depression, who had never smoked and drunk, and with a normal weight (body mass index: 18.5–23.9 kg/m^2^).

**Conclusion:** The cognitive function of actively treated diabetic patients was better than that of patients without diabetes, but the improvement was significant only in elderly people. Depression, smoking, drinking, and an abnormal weight may attenuate this effect.

## Introduction

Approximately 463 million people live with diabetes mellitus (DM) worldwide (Lancet, 2017; Federation I.D., 2019; Li et al., 2020). It is considered as “the third brain disease” and presents considerable threat by exacerbating glomerular microcirculation, arteriosclerosis, retinopathy, metabolic abnormalities and neuropathy and has, thus, become a public health issue of global concern (Zheng et al., 2018). In China, the prevalence of diabetes has reached 30.2% in people aged 60 or older and is increasing yearly as the process of ageing society of China accelerates (Li et al., 2020). Dementia is a global health challenge owing to its destructive effects on the brain (Wortmann, 2012), and its prevalence has reached 6.0% in adults aged 60 or older in China (Jia et al., 2020). DM and dementia have caused huge economic losses and medical burdens in China (Xu et al., 2017; Liu et al., 2019). Given that DM is a booster for dementia, DM treatment and its relationship with dementia is worthy of special attention in China (Biessels et al., 2006; Frankish and Horton, 2017; Callisaya et al., 2019; Marseglia et al., 2019).

High glycaemia can cause vascular injury and affect blood flow (Lyu et al., 2020). Insulin resistance, a frequent symptom of DM, can increase β-amyloid levels and induce the hyperphosphorylation of the tau protein (Biessels and Despa, 2018; Lyu et al., 2020). Nevertheless, patients with DM undergoing active treatment have lower abnormally aggregated protein levels and lighter vascular injuries than those without treatment (McIntosh and Nation, 2019; van Sloten et al., 2020; Yaribeygi et al., 2020). In addition, metformin, an insulin sensitiser, can significantly reduce the rate of cognitive decline and the risk of dementia in patients with DM (Samaras et al., 2020). Using one or more diabetes medications, such as biguanides and sulfonylureas, changes the link between tau pathology and DM and slows down the progression of cognitive decline (McIntosh and Nation, 2019). Therefore, the active treatment of diabetes has been recommended for the prevention of dementia.

As a progressive disease, a long period of cognitive decline occurs before dementia (Ma et al., 2015; Pal et al., 2018). Strengthening the early prevention of cognitive impairment is the most effective measure to control the onset of dementia. Not only elderly people but also middle-aged people with DM show poorer cognitive performance and faster cognitive decline (Tuligenga et al., 2014). A retrospective study suggested that the association between DM and Alzheimer's disease (AD) is stronger in middle-aged people than in elderly people (Smolina et al., 2015), implying that age plays an important role in the relationship between DM and AD. Regardless of age, there are always subjects who develop diabetes but are not actively treated. However, whether or not diabetes treatment exerts the same cognitive benefits in middle-aged and older adults is unclear.

In this study, we attempted to evaluate the relationship between diabetes treatment behaviour and cognition in middle-aged and elderly adults and further explore the impact of age-related risk factors on the relationship between diabetes treatment behaviour and cognitive decline to evoke aggressive treatment behaviours in diabetes patients.

## Materials and Methods

### Study Population

Data were collected from the Chinese Health and Retirement Longitudinal Study (CHARLS), a nationally representative survey on a community-based population aged ≥45 years conducted by the National School of Development at the Peking University. CHARLS recruit subjects with a multistage probability sampling procedure, covering 450 communities through three waves. In total, the baseline survey enrolled 17,708 individuals from 2011 to 2012 (wave 1), 15,770 people were reinterviewed in the second wave (2013–2014, wave 2) and 13,002 people were reinterviewed in the third wave (2015–2016, wave 3). Questionnaire investigation, blood sample collection and anthropometric measurements were included in the surveys.

We selected participants in wave 1 and excluded (1) participants with memory-related disease (*n* = 277) or brain damage (*n* = 450) in wave 1 and (2) participants without information on diabetes (*n* = 133) and cognitive function in wave 1 (*n* = 3,157). Eventually, 13,691 participants were included in this study. We divided participants into <60 years and ≥60 years groups.

### Definition of Diabetes Status

After an overnight fast, the venous blood samples of participants were collected by medically trained staff from the Chinese Center for Disease Prevention and Control (CDC). The blood samples were separated into plasma and buffy coats, immediately stored and frozen at −20°C and transported to the Chinese CDC within 2 weeks, where they were placed in a deeper freezer and stored at −80°C until assay at the Capital Medical University laboratory. Fasting plasma glucose was measured using an enzymatic colorimetric test, whereas glycated haemoglobin (HbA1c) was measured using boronate affinity high-performance liquid chromatography.

The diabetes status was divided into three groups, namely, diabetes-free, treated diabetes and untreated diabetes. The definition of diabetes was based on self-reported physician diagnosis or fasting blood glucose (FBG) or HbA1c measurement (FBG ≥ 126 mg/dl or HbA1c ≥ 6.5%) according to the American Diabetes Association criteria (Association AD, 2019). The questionnaire asked “Do you take any treatment (including insulin injections, Western modern medicine and Chinese traditional medicine) to control your diabetes?” Since previous studies have shown that insulin therapy (Plastino et al., 2010; Avgerinos et al., 2018; Lyu et al., 2020), Western modern medicine such as metformin, sulfonylureas and dipeptidyl peptidase-4 inhibitor (Lyu et al., 2020; Samaras et al., 2020) and Chinese traditional medicine (Xu et al., 2013; Seto et al., 2015) were associated with better cognitive function and lower the risk of dementia, we regarded the three kinds of treatment as effective in this study. Participants with diabetes who answered “Yes” were assigned to the treated-diabetes group, whereas those who answered “No” were assigned to the untreated-diabetes group.

### Measurement of Cognitive Function

Cognitive function was assessed by two measurements, namely, episodic memory and mental intactness. Episodic memory was evaluated by immediate word recall and delayed recall. Interviewers read a list consisting of 10 Chinese words and asked participants to repeat as many words as they could remember. After 5 min, the participants were required to recall the same word list. The number of correctly recalled words was scored in both tests, and the final score of episodic memory was calculated as the sum of immediate and delayed word recall scores (ranging from 0 to 20). Mental intactness consisted of time orientation (awareness of date of today, including year, month, week, day and season), numerical ability (subtracting 7 from 100, up to five times) and picture drawing (replicate a picture shown to them as similar as possible). The score of mental intactness was based on the number of correct answers, ranging from 0 to 11. According to previous studies (Zhou et al., 2020; Liu et al., 2021), we identified global cognition as the summation of episodic memory and mental intactness scores, and high scores reflect good cognitive function.

### Covariates

Covariates included age, sex, education level, marital status, body mass index (BMI), smoking status, drinking status, hypertension, dyslipidaemia, heart disease, stroke and depression. Education level was allocated into primary school or below, middle school/high school and college or above. Marital status was defined as married or unmarried. Smoking and drinking status were categorised into never smoke/drink, former smoker/drinker and current smoking/drinking groups. BMI was calculated as weight in kilogramme divided by height squared in metres and categorised into four groups according to China criteria (Qian et al., 2017): <18.5 kg/m^2^, 18.5–23.9 kg/m^2^, 24–27.9 kg/m^2^ and ≥ 28 kg/m^2^. Hypertension was determined by self-reported doctor diagnosis and baseline blood pressure, and systolic blood pressure of ≥140 mmHg or diastolic blood pressure of ≥90 mmHg was defined as hypertension. Dyslipidaemia was identified by self-reported diagnosis and baseline blood lipid level (total cholesterol ≥240 mg/dl, low-density lipoprotein cholesterol >160 mg/dl, triglycerides ≥200 mg/dl or high-density lipoprotein cholesterol <40 mg/dl). Heart disease and stroke were determined by the self-reported conditions in the questionnaire. Depression was measured using the 10-item Center for Epidemiologic Studies Depression scale-short form (Yang et al., 2020). The scores ranged from 0 to 30. We used a cutoff point of 12 to generate a binary depression variable (no and yes) according to a previous study (Cheng and Chan, 2005).

### Statistical Analysis

The characteristics of the respondents with different diabetes statuses were described using descriptive statistics by age groups. Continuous variables were presented as mean with standard deviation, and one-way ANOVA was used in comparing differences between diabetes status groups. Categorical variables were presented by the frequency with percentage, and the chi-square test was used for description.

Multivariable linear models were used in examining the association between diabetes status and cognitive function. To explore the influential factors contributing to the main findings, we used sex, educational level, marital status, hypertension, dyslipidaemia, heart disease, stroke, BMI, smoking status and drinking status as subgroups to evaluate the relationship between diabetes status and cognitive function. Multiple imputation by chained equations was used to replace missing data in covariates. We completed our statistical analysis using STATA 15.0, StataCorp LLC, College Station, Texas and statistical significance was defined as two-tailed *p* < 0.05.

## Results

### Characteristics of the Study Population

The mean age of the 13,691 participants was 58.55 ± 9.64 years, and 47.40% of the participants were men. The treated-diabetes group had a higher mean age than the other two diabetes status groups in a middle-aged population, and no significant difference in mean age was found among the groups in the elderly population. High proportions of hypertension, heart disease, stroke, dyslipidaemia, overweight, obesity, current smoker and current drinker were observed in the treated-diabetes groups of the two age groups (Table 1).

**Table 1:** Characteristics of the study population among two age groups.

**Variables**	**Diabetes-free**	**Treated-diabetes**	**Untreated diabetes**	***P***	**Diabetes-free**	**Treated-diabetes**	**Untreated diabetes**	***P***
	** <60 years**				**≥60 years**			
*n*	7,036	250	628		4,928	304	545	
Age (years)	51.75 (4.81)	53.35 (4.35)	52.05 (4.64)	<0.001	67.71 (6.48)	67.44 (6.44)	68.34 (6.66)	0.065
Male (*n*, %)	3,373 (47.94)	123 (49.20)	284 (45.22)	0.383	2,348 (47.65)	131 (43.09)	230 (42.20)	0.021
Educational level (*n*, %)	
Primary school and below	3,872 (55.03)	127 (50.80)	321 (51.11)	0.182	3,927 (79.69)	193 (63.49)	445 (81.65)	<0.001
Middle school	2,975 (42.28)	115 (46.00)	293 (46.66)		897 (18.20)	94 (30.92)	90 (16.51)	
College and above	189 (2.69)	8 (3.20)	14 (2.23)		104 (2.11)	17 (5.59)	10 (1.83)	
Married (*n*, %)	6,949 (94.50)	235 (94.00)	596 (94.90)	0.854	3,956 (80.28)	262 (86.18)	429 (78.72)	0.024
Hypertension (*n*, %)	1,214 (17.25)	120 (48.00)	157 (25.00)	<0.001	1,418 (28.77)	193 (63.49)	224 (41.10)	<0.001
Heart disease (*n*, %)	589 (8.37)	52 (20.80)	75 (11.94)	<0.001	732 (14.85)	98 (32.24)	95 (17.43)	<0.001
Stroke (*n*, %)	73 (1.04)	10 (4.00)	9 (1.43)	0.001	117 (2.37)	25 (8.22)	15 (2.75)	<0.001
Dyslipidaemia (*n*, %)	509 (7.23)	79 (31.60)	92 (14.65)	<0.001	441 (8.95)	128 (42.11)	77 (14.13)	<0.001
Depression (*n*, %)	2,091 (29.72)	89 (35.60)	220 (35.03)	0.004	1,693 (34.35)	119 (39.14)	186 (34.13)	0.228
BMI (kg/m^2^)								
<18.5	305 (4.33)	5 (2.00)	28 (4.46)	<0.001	478 (9.70)	8 (2.63)	41 (7.52)	<0.001
18.5~23.9	3,684 (52.36)	89 (35.60)	269 (42.83)		2,714 (55.07)	109 (35.53)	268 (49.17)	
24~27.9	2,304 (32.75)	92 (36.80)	226 (35.99)		1,322 (26.83)	135 (44.41)	172 (31.56)	
≥28	743 (10.56)	64 (25.60)	105 (16.72)		414 (8.40)	53 (14.73)	64 (11.74)	
Smoking (*n*, %)								
Never	4,321 (61.41)	156 (62.40)	397 (63.22)	0.001	2,929 (59.44)	204 (67.11)	336 (61.65)	<0.001
Former	2,238 (31.81)	61 (24.40)	185 (29.46)		1,458 (29.59)	53 (17.43)	154 (28.26)	
Current	477 (6.78)	33 (13.20)	46 (7.32)		541 (10.98)	47 (15.46)	55 (10.09)	
Drinking (*n*, %)								
Never	4,115 (58.48)	145 (58.00)	352 (56.05)	<0.001	2,933 (59.52)	205 (67.43)	331 (60.73)	<0.001
Former	2,529 (35.94)	73 (29.20)	235 (37.42)		1,472 (29.87)	52 (17.11)	142 (26.06)	
Current	392 (5.57)	32 (12.80)	41 (6.53)		523 (10.61)	47 (15.46)	72 (13.21)	

### Cognitive Differences in Different Diabetes Status

**Figure 1:**
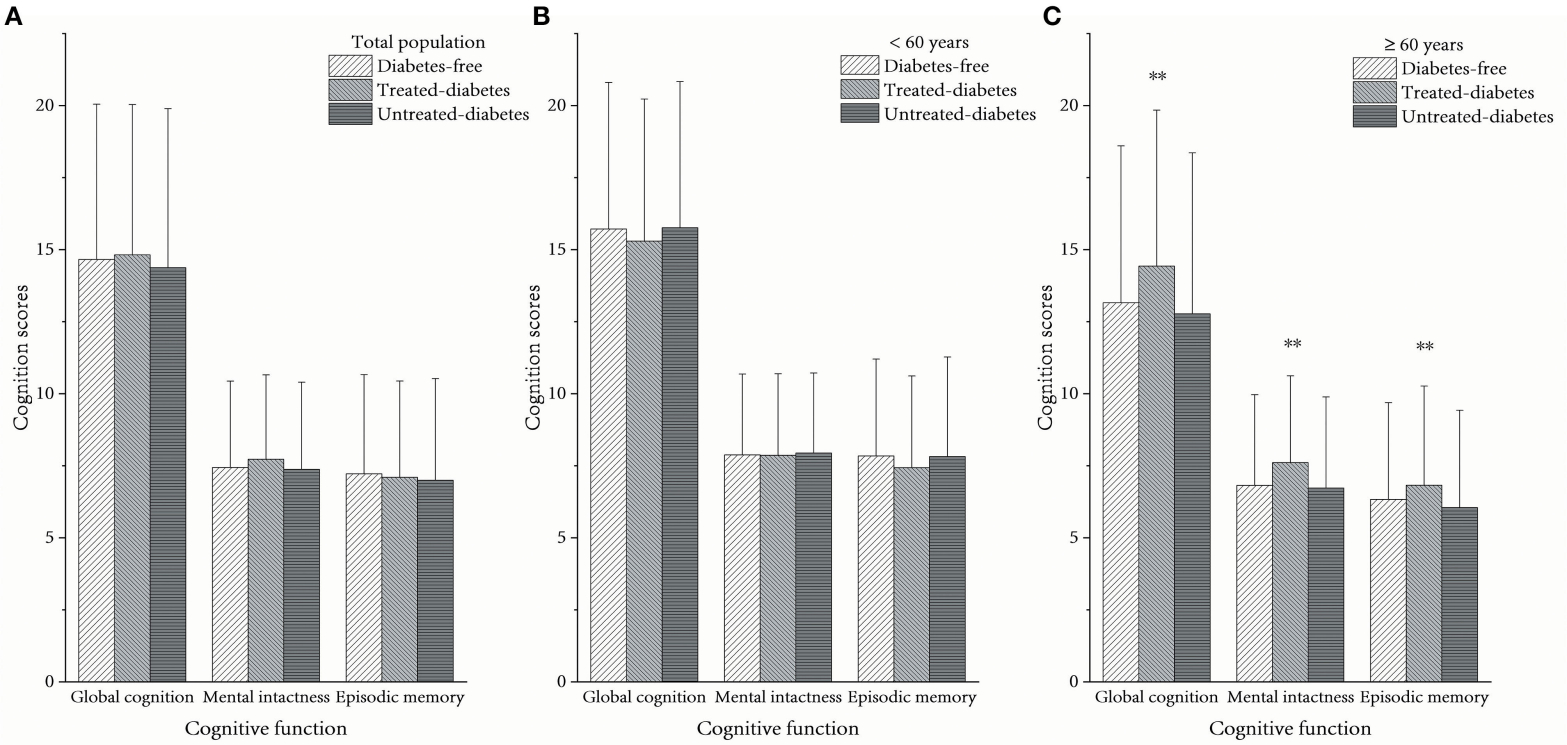
Average cognition scores between different diabetes statuses. **(A)** In the total population, **(B)** in the population aged <60 years, and **(C)** in the population aged ≥60 years. ^**^The scores among the three groups were significantly different as *p* < 0.05.

The cognition scores of global cognition and mental intactness in the treated-diabetes group in the whole population were slightly higher than those in the other groups. All kinds of cognition scores in the treated-diabetes group comprising individuals aged >60 were significantly higher than those in the other two groups [global cognition: *p* = 0.0001, η^2^ = 0.0033 (95% CI = 0.0010–0.0067); mental intactness: *p* = 0.0001, η^2^ = 0.0033 (95% CI = 0.0010–0.0067); episodic memory: *p* = 0.0055, η^2^ = 0.0018 (95% CI = 0.0002–0.0044); Figure 1].

### Association Between Diabetes Status and Cognitive Function

Multivariable linear regression showed that treated diabetes was positively associated with mental intactness in all participants (β = 0.27, 95% CI = 0.04–0.50) and participants aged ≥60 (β = 0.44, 95% CI = 0.12–0.77) after adjustment for age, sex, educational level, marital status, smoking status and drinking status (Table 2). However, the results of mental intactness in all participants lost significance, but the significance remained in the elderly after further adjustment for BMI, hypertension, dyslipidaemia, heart disease, stroke and depression. Diabetes status was not associated with cognition in middle-aged subjects.

**Table 2:** Association between diabetes status and cognitive function, using the diabetes-free group as reference.

**Diabetes status**	**All participants**		**<** **60 years**		**≥** **60 years**	
	**β (95% CI)**	***P***	**β (95% CI)**	***P***	**β (95% CI)**	***P***
**Model 1**						
**Global cognition**						
Treated-diabetes	0.21 (−0.20,0.62)	0.311	−0.37 (−0.97,0.23)	0.223	0.54 (−0.03,1.10)	0.063
Untreated-diabetes	−0.03 (−0.32,0.25)	0.821	0.02 (−0.36,0.41)	0.905	−0.05 (−0.48,0.38)	0.804
**Mental intactness**						
Treated-diabetes	0.27 (0.04,0.50)	0.022	−0.03 (−0.36,0.30)	0.852	0.44 (0.12,0.77)	0.008
Untreated-diabetes	0.07 (−0.09,0.23)	0.412	0.06 (−0.15,0.27)	0.596	0.10 (−0.14,0.35)	0.412
**Episodic memory**						
Treated-diabetes	−0.06 (−0.33,0.22)	0.678	−0.34 (−0.75,0.07)	0.104	0.09 (−0.27,0.46)	0.621
Untreated-diabetes	−0.10 (−0.29,0.09)	0.303	−0.03 (−0.30,0.23)	0.799	−0.16 (−0.44,0.12)	0.265
**Model 2**						
**Global cognition**						
Treated-diabetes	0.16 (−0.25,0.57)	0.446	−0.35 (−0.95,0.24)	0.246	0.43 (−0.14,1. 00)	0.139
Untreated-diabetes	−0.05 (−0.34,0.23)	0.716	0.06 (−0.32,0.44)	0.750	−0.14 (−0.56,0.29)	0.528
**Mental intactness**						
Treated-diabetes	0.22 (−0.01,0.45)	0.063	−0.04 (−0.37,0.29)	0.793	0.37 (0.04,0.70)	0.029
Untreated-diabetes	0.05 (−0.11,.0.21)	0.555	0.07 (−0.14,0.28)	0.537	0.05 (−0.20,0.30)	0.686
**Episodic memory**						
Treated-diabetes	−0.06 (−0.34,0.22)	0.668	−0.31 (−0.72,0.10)	0.141	0.06 (−0.31,0.43)	0.744
Untreated-diabetes	−0.10 (−0.29,0.09)	0.299	−0.005 (−0.27,0.26)	0.972	−0.19 (−0.47,0.09)	0.185

*Model 1: adjusted for age, sex, educational level, marital status, smoking status and drinking status*.

*Model 2: adjusted for model 1 + BMI, hypertension, dyslipidaemia, heart disease, stroke and depression*.

Subgroup analysis showed that being underweight was associated with poor global cognition performance [β = −2.17, 95% CI = –(4.18–0.15)] and episodic memory performance [β = −1.51, 95% CI = –(2.84–0.19)] in middle-aged people in the untreated-diabetes group. Meanwhile, middle-aged people in the treated-diabetes group who were overweight [β = −0.67, 95% CI = –(1.32–0.01)], former smokers [β = −1.27, 95% CI = –(2.51–0.04)] and drinkers [β = −0. 75, 95% CI = –(1.49–0.01)] had poor cognitive functions (Table 3). Moreover, elderly people in the treated-diabetes group without depression (β = 0.43, 95% CI = 0.02–0.85), without smoking history (β = 0.51, 95% CI = 0.09–0.93) and without drinking history (β = 0.42, 95% CI = 0.01–0.83) and those with normal weight (BMI: 18.5–23.9 kg/m^2^; β = 0.59, 95% CI = 0.05–1.13) were associated with good mental intactness (Table 4).

**Table 3:** Relationships between diabetes status (diabetes-free as the reference group) and cognitive function in the subgroups (< 60 years).

**Subgroups**	**Global cognition**			**Mental intactness**			**Episodic memory**		
	**Treated-diabetes**	**Untreated-diabetes**	***P*** **for interaction^a^**	**Treated-diabetes**	**Untreated-diabetes**	***P*** **for interaction**	**Treated-diabetes**	**Untreated-diabetes**	***P*** **for interaction**
	**β (95% CI)**	**β (95% CI)**		**β (95% CI)**	**β (95% CI)**		**β (95% CI)**	**β (95% CI)**	
**<60 years**									
Gender			0.301			0.541			0.313
Male	−0.51 (−1.35, 0.33)	0.18 (−0.36, 0.72)		−0.06 (−0.53, 0.40)	0.08 (−0.21, 0.38)		−0.45 (−1.05, 0.14)	0.09 (−0.29, 0.48)	
Female	0.002 (−0.91, 0.92)	−0.19 (−0.75, 0.37)		0.05 (−0.46, 0.57)	−0.02 (−0.34, 0.29)		−0.05 (−0.67, 0.57)	−0.17 (−0.55, 0.21)	
Education			0.800			0.983			0.725
Primary school or below	−0.88 (−1.74, −0.02)^*^	0.04 (−0.52, 0.60)		−0.19 (−0.70, 0.33)	0.12 (−0.20, 0.45)		−0.37 (−0.95, 0.22)	−0.08 (−0.45, 0.29)	
Middle school/high school	0.16 (−0.74, 1.06)	−0.07 (−0.62, 0.49)		0.27 (−0.20, 0.74)	−0.06 (−0.35, 0.23)		−0.11 (−0.76, 0.55)	−0.01 (−0.41, 0.40)	
College or above	−0.33 (−4.52, 3.86)	0.43 (−1.63, 2.49)		0.01 (−2.00, 2.02)	0.23 (−0.75, 1.22)		−0.34 (−3.96, 3.27)	0.19 (−1.58, 1.97)	
Marital status			0.127			0.958			0.024
No	−0.79 (−3.30, 1.73)	1.05 (−0.79, 2.88)		−0.60 (−2.10, 0.91)	−0.19 (−1.28, 0.91)		−0.19 (−1.95, 1.57)	1.23 (−0.05, 2.51)	
Yes	0.26 (−0.91, 0.38)	−0.07 (−0.47, 0.33)		0.01 (−0.34, 0.37)	0.05 (−0.18, 0.27)		−0.28 (−0.72, 1.27)	−0.12 (−0.40, 0.15)	
Hypertension			0.056			0.107			0.140
No	−0.23 (−1.08, 0.63)	−0.30 (−0.75, 0.15)		0.21 (−0.27, 0.68)	−0.12 (−0.37, 0.14)		−0.43 (−1.02, 0.15)	−0.19 (−0.50, 0.13)	
Yes	−0.20 (−1.11, 0.70)	0.76 (0.01, 1.51)		−0.19 (−0.71, 0.32)	0.46 (0.03, 0.89)		−0.01 (−0.63, 0.61)	0.35 (−0.18, 0.88)	
Dyslipidaemia			0.206			0.625			0.149
No	−0.43 (−1.19, 0.32)	−0.02 (−0.44, 0.40)		−0.07 (−0.50, 0.35)	0.05 (−0.18, 0.29)		−0.36 (−0.88, 0.16)	−0.07 (−0.36, 0.22)	
Yes	−0.11 (−1.20, 0.98)	−0.05 (−1.10, 1.00)		0.09 (−0.53, 0.70)	−0.003 (−0.59, 0.59)		−0.20 (−0.96, 0.56)	−0.04 (−0.77, 0.68)	
Heart disease			0.485			0.816			0.229
No	−0.14 (−0.84, 0.55)	−0.07 (−0.49, 0.34)		0.12 (−0.26, 0.51)	0.04 (−0.19, 0.27)		−0.27 (−0.74, 0.21)	−0.11 (−0.40, 0.17)	
Yes	−0.68 (−2.13, 0.76)	0.52 (−0.63, 1.66)		−0.55 (−1.38, 0.27)	0.07 (−0.58, 0.73)		−0.13 (−1.12, 0.86)	0.44 (−0.34, 1.23)	
Stroke			0.106			0.221			0.175
No	−0.14 (−0.77, 0.50)	0.03 (−0.37, 0.42)		0.05 (−0.31, 0.40)	0.06 (−0.16, 0.28)		−0.18 (−0.62, 0.25)	−0.04 (−0.31, 0.24)	
Yes	−3.17 (−6.45, 0.10)	−1.76 (−4.92, 1.41)		−0.84 (−2.68, 1.00)	−0.91 (−2.69, 0.87)		−2.34 (−4.82, 0.15)	−0.85 (−3.25, 1.56)	
Depression			0.969			0.940			0.905
No	−0.31 (−1.08, 0.47)	0.004 (−0.48, 0.58)		0.03 (−0.40, 0.46)	0.03 (−0.24.0.29)		−0.34 (−0.88, 0.21)	−0.02 (−0.36, 0.31)	
Yes	0.10 (−0.95, 1.15)	−0.01 (−0.69, 0.66)		0.06 (−0.56, 0.67)	0.10 (−0.29, 0.50)		0.04 (−0.65, 0.74)	−0.12 (−0.56, 0.33)	
BMI			0.455			0.554			0.545
<18.5	−0.65 (−5.23, 3.93)	−2.17 (−4.18, −0.15)^*^		1.12 (−1.51, 3.76)	−0.65 (−1.81, 0.51)		−1.77 (−4.79, 1.24)	−1.51 (−2.84, −0.19)^*^	
18.5~23.9	−0.50 (−1.34, 0.35)	−0.19 (−0.70, 0.33)		0.01 (−0.47, 0.49)	−0.06 (−0.35, 0.23)		−0.72 (−1.39, −0.05)	−0.13 (−0.48, 0.22)	
24~27.9	0.25 (−0.88, 1.38)	0.40 (−0.29, 1.10)		−0.67 (−1.32, −0.01)^*^	0.26 (−0.13, 0.64)		0.02 (−0.77, 0.81)	0.15 (−0.34, 0.63)	
≥28	0.06 (−1.57, 1.70)	−0.004 (−1.20, 1.20)		−0.22 (−1.11, 0.67)	0.11 (−0.54, 0.76)		0.28 (−0.87, 1.42)	−0.12 (−0.96, 0.72)	
Smoking			0.487			0.346			0.808
Never	0.06 (−0.75, 0.86)	−0.18 (−0.69, 0.33)		0.14 (−0.31, 0.59)	−0.05 (−0.33, 0.24)		−0.08 (−0.63, 0.46)	−0.13 (−0.48, 0.21)	
Former	−1.27 (−2.51, −0.04)^*^	0.36 (−0.31, 1.04)		−0.52 (−1.21, 0.17)	0.19 (−0.19, 0.56)		−0.75 (−1.62, 0.11)	0.18 (−0.30, 0.65)	
Current	0.01 (−1.66, 1.68)	−0.32 (−1.73, 1.09)		0.28 (−0.64, 1.20)	0.08 (−0.70, 0.86)		−0.26 (−1.45, 0.92)	−0.40 (−1.40, 0.60)	
Drinking			0.789			0.817			0.564
Never	0.14 (−0.70, 0.97)	0.04 (−0.49, 0.58)		0.14 (−0.33, 0.61)	0.05 (−0.25, 0.36)		0.001 (−0.57, 0.57)	−0.01 (−0.38, 0.36)	
Former	−0.99 (−2.11, 0.13)	−0.14 (−0.74, 0.47)		−0.21 (−0.83, 0.41)	−0.09 (−0.42, 0.25)		−0.75 (−1.49, −0.01)^*^	−0.05 (−0.48, 0.37)	
Current	−0.40 (−2, 16, 1.36)	0.26 (−1.31, 1.84)		−0.20 (−1.20, 0.81)	0.63 (−0.27, 1.53)		−0.20 (−1.36, 0.97)	−0.37 (−1.41, 0.68)	

*Adjusted for age, sex, educational level, marital status, hypertension, dyslipidaemia, heart disease, stroke, depression, BMI, smoking status and drinking status*.

* *p < 0.05*.

a *The interaction is an interaction term between each subgroup variable and diabetes status*.

**Table 4:** Relationships between diabetes status (diabetes-free as the reference group) and cognitive function in the subgroups (≥60 years).

**Subgroups**	**Global cognition**			**Mental intactness**			**Episodic memory**		
	**Treated-diabetes**	**Untreated-diabetes**	***P*** **for interaction^a^**	**Treated-diabetes**	**Untreated-diabetes**	***P*** **for interaction**	**Treated-diabetes**	**Untreated-diabetes**	***P*** **for interaction**
	**β (95% CI)**	**β (95% CI)**		**β (95% CI)**	**β (95% CI)**		**β (95% CI)**	**β (95% CI)**	
Gender			0.214			0.204			0.435
Male	0.58 (−0.30, 1.46)	−0.33 (−0.99, 0.32)		0.39 (−0.12, 0.90)	−0.12 (−0.50, 0.27)		0.19 (−0.41, 0.78)	−0.22 (−0.66, 0.23)	
Female	0.44 (−0.42, 1.30)	0.08 (−0.55, 0.70)		0.34 (−0.16, 0.83)	0.12 (−0.24, 0.48)		0.10 (−0.45, 0.65)	−0.04 (−0.44, 0.36)	
Education			0.330			0.733			0.235
Primary school or below	0.32 (−0.46, 1.10)	−0.23 (−0.74, 0.28)		0.39 (−0.08, 0.85)	−0.02 (−0.31, 0.28)		−0.07 (−0.57, 0.43)	−0.21 (−0.54, 0.11)	
Middle school/high school	1.01 (0.05, 1.97)^*^	0.15 (−0.90, 1.20)		0.39 (−0.16, 0.94)	0.09 (−0.48, 0.66)		0.69 (−0.04, 1.42)	0.06 (−0.70, 0.81)	
College or above	−1.25 (−4.14, 1.64)	2.07 (−1.38, 5.51)		−0.75 (−2.04, 0.55)	0.13 (−1.41, 1.67)		−0.50 (−2.82, 1.81)	1.94 (−0.82, 4.70)	
Marital status			0.193			0.213			0.374
No	−0.11 (−1.76, 1.55)	0.25 (−0.75, 1.25)		0.55 (−0.41, 1.52)	−0.001 (−0.58, 0.58)		−0.67 (−1.74, 0.42)	0.25 (−0.40, 0.90)	
Yes	0.60 (−0.06, 1.27)	−0.22 (−0.73, 0.28)		0.38 (0.02, 0.73)^*^	0.01 (−0.28, 0.30)		0.25 (−0.18, 0.69)	−0.23 (−0.56, 0.10)	
Hypertension			0.299			0.510			0.315
No	0.26 (−0.73, 1.24)	−0.28 (−0.86, 0.31)		0.36 (−0.21, 0.93)	−0.03 (−0.37, 0.30)		−0.10 (−0.75, 0.55)	−0.24 (−0.63, 0.14)	
Yes	0.64 (−0.15, 1.42)	0.15 (−0.56, 0.87)		0.35 (−0.11, 0.81)	0.07 (−0.34, 0.49)		0.28 (−0.22, 0.79)	0.08 (−0.38, 0.54)	
Dyslipidaemia			0.313			0.637			0.262
No	0.57 (−0.24, 1.37)	−0.19 (−0.67, 0.30)		0.42 (−0.05, 0.89)	0.01 (−0.28, 0.29)		0.15 (−0.38, 0.68)	−0.19 (−0.51, 0.13)	
Yes	0.14 (−0.88, 1.16)	0.40 (−0.85, 1.65)		0.10 (−0.48, 0.69)	0.09 (−0.63, 0.81)		0.03 (−0.63, 0.69)	0.31 (−0.51, 1.12)	
Heart disease			0.060			0.010			0.555
No	0.40 (−0.34, 1.14)	−0.32 (−0.81, 0.18)		0.38 (−0.04, 0.81)	−0.15 (−0.44, 0.14)		0.01 (−0.47, 0.50)	−0.17 (−0.49, 0.16)	
Yes	0.71 (−0.43, 1.84)	0.79 (−0.33, 1.91)		0.29 (−0.36, 0.95)	0.87 (0.26, 1.47)		0.41 (−0.30, 1.13)	0.001 (−0.71, 0.71)	
Stroke			0.121			0.070			0.442
No	0.33 (−0.31, 0.97)	−0.13 (−0.59, 0.32)		0.30 (−0.07, 0.67)	−0.01 (−0.28, 0.25)		0.04 (−0.38, 0.45)	−0.12 (−0.42, 0.18)	
Yes	2.32 (0.03, 4.63)^*^	1.19 (−1.87, 4.24)		0.95 (−0.57, 2.47)	1.31 (−0.61, 3.22)		1.37 (−0.12, 2.87)	−0.12 (−2.03, 1.79)	
Depression			0.292			0.121			0.813
No	0.63 (−0.16, 1.42)	0.01 (−0.56, 0.57)		0.43 (0.02, 0.85)^*^	0.15 (−0.18, 0.47)		0.23 (−0.30, 0.76)	−0.14 (−0.52, 0.24)	
Yes	0.28 (−0.69, 1.25)	−0.36 (−1.11, 0.38)		0.26 (−0.33, 0.85)	−0.26 (−0.71, 0.20)		0.02 (−0.60, 0.64)	−0.11 (−0.58, 0.37)	
BMI			0.296			0.394			0.401
<18.5	1.55 (−1.77, 4.88)	1.01 (−0.52, 2.55)		1.54 (−0.43, 3.50)	0.81 (−0.10, 1.71)		0.02 (−2.15, 2.19)	0.20 (−0.80, 1.20)	
18.5~23.9	0.78 (−0.05, 1.62)	−0.29 (−0.86, 0.28)		0.59 (0.05, 1.13)^*^	−0.08 (−0.41, 0.25)		0.26 (−0.28, 0.81)	−0.39 (−0.78, −0.001)	
24~27.9	0.05 (−1.01, 1.11)	0.06 (−0.81, 0.93)		0.04 (−0.56, 0.64)	0.16 (−0.33, 0.65)		0.01 (−0.69, 0.71)	−0.09 (−0.67, 0.48)	
≥28	0.57 (−1.34, 2.49)	0.58 (−0.96, 2.11)		0.47 (−0.64, 1.57)	0.28 (−0.61, 1.16)		0.11 (−1.10, 1.31)	0.30 (−0.67, 1.27)	
Smoking			0.852			0.802			0.611
Never	0.48 (−0.30, 1.26)	−0.25 (−0.84, 0.34)		0.51 (0.09, 0.93)^*^	−0.01 (−0.35, 0.33)		0.02 (−0.49, 0.52)	−0.24 (−0.62, 0.14)	
Former	0.68 (−0.72, 2.08)	0.27 (−0.54, 1.07)		0.24 (−0.58, 1.07)	0.06 (−0.42, 0.53)		0.44 (−0.49, 1.36)	0.21 (−0.32, 0.75)	
Current	0.11 (−1.39, 1.61)	−0.23 (−1.71, 1.24)		−0.06 (−0.90, 0.79)	0.14 (−0.69, 0.98)		0.17 (−0.86, 1.20)	−0.38 (−1.39, 0.64)	
Drinking			0.632			0.562			0.827
Never	0.42 (−0.36, 1.20)	−0.07 (−0.66, 0.53)		0.42 (0.01, 0.83)^*^	0.05 (−0.29, 0.39)		0.12 (−0.39, 0.62)	−0.12 (−0.50, 0.27)	
Former	0.94 (−0.42, 2.30)	−0.06 (−0.90, 0.78)		0.48 (−0.27, 1.23)	0.01 (−0.48, 0.49)		0.07 (−0.86, 1.00)	−0.07 (−0.64, 0.50)	
Current	0.40 (−1.16, 1.97)	−0.48 (−1.74, 0.78)		0.03 (−0.89, 0.96)	−0.16 (−0.91, 0.58)		0.37 (−0.63, 1.37)	−0.31 (−1.12, 0.49)	

*Adjusted for age, sex, educational level, marital status, hypertension, dyslipidaemia, heart disease, stroke, depression, BMI, smoking status and drinking status*.

* *p < 0.05*.

a *The interaction is an interaction term between each subgroup variables and diabetes status*.

## Discussion

In this study, we selected the diabetes-free group as the reference group. We found that patients with diabetes had greater cognitive gains from active treatment than those without diabetes, but significant gains were observed only in elderly adults. The results were more obvious in patients without depression and those who never smoked and drunk and had normal weights (BMI 18.5–23.9 kg/m^2^).

Antidiabetic agents have anti-inflammatory, antioxidant and anti-autophagy effects (Yaribeygi et al., 2020), which are the potential mechanisms against the neurodegenerative complications of diabetes and other neurological disorders. The inflammatory hypothesis has been proposed in the pathology of diabetes complications. Antidiabetic agents can suppress the expression of inflammatory mediators and thereby alleviate inflammatory responses (Yaribeygi et al., 2019a). Diabetes disrupts the physiologic redox state, therefore promoting an increase in free radicals and the development of oxidative stress, which contribute to vascular complications. Antidiabetic medications may protect microcirculation and macrocirculation mainly through their hypoglycemic effect, reducing glucose-induced free radical generation (Yaribeygi et al., 2019b). Antidiabetic agents can also induce autophagy pathways and modulate this process, improving cognitive function (Ashrafizadeh et al., 2019).

Diabetes is a risk factor for dementia both in midlife and late-life people (Tuligenga et al., 2014; Marseglia et al., 2019). Some studies demonstrated that the effect of diabetes on dementia risk decreases with age, suggesting that the age of exposure to diabetes is related to the development of dementia (Xu et al., 2009; Cheng et al., 2012; Smolina et al., 2015; Barbiellini et al., 2021). As previously reported, diabetes onset in midlife was associated with a higher risk of dementia compared with late-life diabetes (Barbiellini et al., 2021). This phenomenon was also found in other cardiovascular risk factors. Recently, some studies indicated that the associations of several cardiovascular risk factors and vascular disorders with the risk of dementia were age-dependent, and these factors were associated with a high risk of dementia in midlife but not necessarily in late-life (Anstey et al., 2014; Legdeur et al., 2019; Olaya et al., 2019). Therefore, elderly people with diabetes are supposed to have better cognitive outcomes than middle-aged people. Our results showed that treatments for diabetes had protective effects on the cognitive function of elderly participants. However, the same phenomenon was not observed in middle-aged people. These findings suggested that the effect of diabetes treatment on the improvement of cognitive function varies with age. The subgroup analysis in this study showed that elderly participants with stroke in the treated-diabetes group were related to better cognitive function, indicating that these patients received greater cognitive benefits from active treatment of diabetes. It is possible that while treating diabetes, cardiovascular disease has also been improved. Moreover, previous studies have shown that some drugs for the treatment of cardiovascular disease also have a protective effect on cognition (Goldstein et al., 2009), so the protective effect was more obvious in these people. However, the mechanisms underlying the age-dependent effect of dementia risk are still unclear, although our study provided a novel insight for further basic research and physiological and pathological research.

According to previous reports, depression increases the risks of dementia and cognitive impairment (Rubin, 2018; Canton-Habas et al., 2020; Yang et al., 2020). In this study, the protective effect of antidiabetic treatment on cognitive function was only observed in elderly individuals without depression. Moreover, depression attenuated the cognitive protective effect of diabetes treatment. Previous studies found that the level of risk of dementia associated with comorbid diabetes and depression was higher than that associated with diabetes alone (Johnson et al., 2015; Black et al., 2018). Depression-related hypothalamic-pituitary axis dysregulation would lead to the chronic elevation of cortisol level and may damage brain regions involved in cognition. Our findings were consistent with those of prior studies and demonstrated that depression plays an adverse role in well-managed diabetes patients. The main reason is that depression is correlated with poor adherence to recommendations for prescription drugs, physical activity and healthy diet in patients with diabetes (Katon et al., 2015; Downer et al., 2016; Lunghi et al., 2017; Choi and Smaldone, 2018). Therefore, preventive measures for depression are essential to the management of diabetes patients.

Overweight, obesity, smoking and drinking are regarded as risk factors for a series of adverse health outcomes, including dementia (Durazzo et al., 2014; Sabia et al., 2014; Ma et al., 2020). Overweight and obesity can increase vascular disorders and thereby cause dementia. The most common measurement of overweight and obesity is BMI, which is a predictor of dementia and cognitive decline (Gustafson et al., 2012). Other researchers suggested that people with dementia have low BMI, and decline in BMI and underweight years before the diagnosis of dementia are related to dementia (Cova et al., 2016; Kang et al., 2021). In this study, the protective effect of diabetes treatment on cognition was observed only in elderly people with normal weights (BMI: 18.5–23.9 kg/m^2^), whereas people who were underweight or overweight had worse cognitive functions, suggesting that low and high BMI are predictors for cognitive decline. Smoking is associated with vascular disorders, which are potential risk factors for dementia and cognitive decline (Durazzo et al., 2014). Moreover, smoking is related to adverse effects on brain neurobiology and function. Such effects cause dementia and mild cognitive impairment. According to subgroup analysis, people who never smoked had better cognition than former smokers. Our results supported the fact that smoking is harmful to cognitive function. Alcohol drinking may affect cognitive function. The subgroup analysis in our study showed that people who never drink can benefit from diabetes treatment, supporting the finding that drinking has adverse effects on cognitive function.

Possible reasons that resulted in the differential effect of diabetes treatment on cognitive function among middle-aged people and elderly people may be the discrepancy of their brain conditions. The brains of middle-aged people may still be resilient enough, so treatment has no effect yet. But the brains of the elderly may be more vulnerable and more sensitive to the treatment of diabetes. Another reason may be that the cognitive decrement of middle-aged patients has not declined in this study, and there is no significant difference among middle-aged people with different diabetes statuses, so the results did not show the effect of treatment.

The principal strength of this study is that it provides a nationally representative sample of community-dwelling middle-aged and older populations in China. The study examined the relationship between diabetes status and cognitive decline in midlife and late-life people and found that diabetes treatment effects vary with age. However, some limitations need to be acknowledged. First, some diabetic patients were unaware of their illnesses in the self-reported questionnaire and were diagnosed using glucose or HbA1c levels. Hence, they were included in the untreated-diabetes group. This situation may have caused misclassification bias. Second, the measurement of cognitive function was based only on the self-reported scales in the questionnaire, and guidance from professional physicians was lacking. Neuropsychological assessment in such a large-scale study is difficult to complete. On account of the limitation of the cognition assessment method in this study, it is difficult to generalise the results to other cognitive domains. Further studies can improve this study by conducting a more integrated and formal neuropsychological assessment. Third, information on medication adherence was insufficient, and some people in the treated-diabetes group may have not taken medications as prescribed. The exact type of antidiabetic medications was unclear. Therefore, which types of medication exerted effects were not identified in this study. Fourth, the covariables HbA1c and FBG were not considered because of the large number of missing data. Individuals without HbA1c and FBG data were categorised only with self-reported diagnosis information. Last, the reason that these people had not been treated for diabetes is unclear. The factors that affected diabetes treatment may have also affected cognition.

In summary, we revealed that diabetes treatment is associated with enhanced cognitive performance in late-life diabetes. However, the same phenomenon was not observed in midlife diabetes, thus warranting further studies. Despite that antidiabetic treatment had a protective effect on participants aged ≥60 years, depression, smoking, drinking and an abnormal BMI may have mitigated this effect.

## Data Availability Statement

Publicly available datasets were analysed in this study. This data can be found here: http://charls.pku.edu.cn/.

## Ethics Statement

The studies involving human participants were reviewed and approved by the Biomedical Ethics Review Committee of Peking University (IRB00001052-11015). The patients/participants provided their written informed consent to participate in this study.

## Author Contributions

KW wrote the article. KW and HL performed the data analysis. HL and JZ drafted and critically revised the manuscript. LZ and SG provided clinical guidance. RZ, ZY, and ZH organised database. XW contributed to the study concept and design and reviewed the article. All authors contributed to the article and approved the submitted version.

## Funding

This work was supported by the National Natural Science Foundation of China (82173607), the Basic and Applied Basic Research Foundation of Guangdong Province (2021A1515011684), the Open Project of the Guangdong Provincial Key Laboratory of Tropical Disease Research (2020B1212060042), and Guangzhou Municipal Science and Technology Project (202102080597).

## Conflict of Interest

The authors declare that the research was conducted in the absence of any commercial or financial relationships that could be construed as a potential conflict of interest.

## Publisher's Note

All claims expressed in this article are solely those of the authors and do not necessarily represent those of their affiliated organizations, or those of the publisher, the editors and the reviewers. Any product that may be evaluated in this article, or claim that may be made by its manufacturer, is not guaranteed or endorsed by the publisher.

## Abbreviations

CHARLS: Chinese Health and Retirement Longitudinal Study
BMI: body mass index
CI: confidence interval
DM: diabetes mellitus
AD: Alzheimer's disease
FBG: fasting blood glucose
HbA1c: glycated haemoglobin.
